# Demethoxycurcumin analogue DMC-BH inhibits orthotopic growth of glioma stem cells by targeting JNK/ERK signaling

**DOI:** 10.18632/aging.103531

**Published:** 2020-07-24

**Authors:** Lei Shi, Guan Sun, Haifeng Zhu

**Affiliations:** 1 Department of Neurosurgery, Affiliated Kunshan Hospital of Jiangsu University, Suzhou 215300, P.R.China; 2 Department of Neurosurgery, The Fourth Affiliated Hospital of Nantong University, Yancheng No.1 People's Hospital, Yancheng 224000, P. R. China; 3 Department of Neurosurgery, Funing People’s Hospital, Funing 224400, P.R.China

**Keywords:** DMC-BH, JNK, ERK, apoptosis, autophagy, proliferation

## Abstract

Glioma stem cells (GSCs) play an important role in glioblastoma resistance to conventional therapies and disease recurrence. Here, we assessed the therapeutic effect of a demethoxycurcumin analogue, DMC-BH, on GSCs, and investigated the underlying mechanisms. Our in vitro data demonstrate that DMC-BH inhibits GSC proliferation, and induces apoptosis and autophagy in GSCs. In addition, our results show that DMC-BH effectively crosses the blood-brain barrier to inhibit the growth of intracranial GSC tumors in vivo. DMC-BH significantly increased phosphorylation levels of JNK, ERK and c-Jun in GSCs. Inhibition of JNK and ERK activities reversed the pro-apoptotic effect of DMC-BH in GSCs, indicating that the DMC-BH-induced apoptosis in GSCs is mediated via the JNK/ERK signaling pathway. These results suggest that DMC-BH could potentially serve as a effective therapy against GSCs that acts by targeting the JNK/ERK signaling pathway.

## INTRODUCTION

Glioma is a common primary brain tumor, accounting for about 45% of all intracranial tumors [1]. Glioblastoma (GBM) is the most aggressive form of brain tumor, with average survival time about 14.6 months [2]. Glioma treatment protocols include surgery, postoperative radiotherapy, and chemotherapy. However, gliomas, and especially glioblastomas frequently relapse; the recurrence rate of glioblastoma is almost 100%. The high recurrence rate of gliomas is associated with the rapid proliferation and invasive growth of glioma cells. Glioma stem cells (GSCs) represent a subpopulation of cells in glioma that have biological characteristics similar to neural stem cells, such as differentiation into neurons, expression of nestin, CD133, and other neural stem cell surface markers [3]. Recent studies have suggested that many established glioma cell lines including U87, U251, GL261, SHG-44, and CHG-5 also have a small number of stem cells [4]. Although GSCs account for only a small proportion of glioma cells, they can proliferate, self-renew, and differentiate, and play a decisive role in the growth and recurrence of glioma. The present treatments of glioma cannot effectively kill GSCs, resulting in the high rates of glioma recurrence. Understanding the mechanisms that regulate GSCs development, survival, and proliferation is crucial for development of therapies to reduce the glioma recurrence.

We have previously found that demethoxycurcumin (DMC), an active compound from the rhizome of Curcuma longa, inhibits the GSC growth and induces apoptosis both in vitro and in vivo [5]. However, DMC has an obvious disadvantage, since it cannot effectively promote its anti-tumor effects through the blood-brain barrier [6]. After optimizing the structure of DMC, we have found an analogue of DMC, named DMC-BH, which can efficiently pass through the blood-brain barrier, and exhibits potent inhibitory effects in orthotopic glioblastomas. However, its function in GSCs is unknown. In this study, our data demonstrate that like DMC, DMC-BH can effectively inhibit GSCs proliferation and induce apoptosis; however, DMC-BH can also inhibit the growth of orthotopic glioblastomas.

Mitogen-activated protein kinases (MAPKs) are a group of conserved serine/threonine protein kinases, including ERK, JNK, and p38 [7]. MAPKs regulate many biological activities, such as inflammation, apoptosis, tumorigenesis, and metastasis [8]. The most widely studied is the ERK1/ERK2 (p42/p44) kinase, which is phosphorylated and activated by MEK1 [9]. Several studies have shown that ERK1/2 activity is increased in gliomas and GSCs [10], and that inhibition of ERK signaling inhibits glioma progression [11]. However, in other studies, inhibition of ERK signaling had the opposite effect. For example, Wang et al. found that Temozolomide (TMZ) inhibited glioma growth by inhibiting the ERK signaling, while curcumin activated the MAPKs/ERK pathway and attenuated the inhibitory effects of TMZ [12]. In human monocytic leukemia THP-1 cells, activation of ERK and JNK signaling played a key role in curcumin-induced apoptosis [13]. JNK, an important member of MAPKs, is activated during apoptosis of glioma cells induced by various substances [14, 15].

In this study, we analyzed the function of DMC-BH in regulating proliferation, apoptosis, and autophagy of GSCs in vitro, and in intracranial orthotopic tumor xenografts in vivo. Our data demonstrate that DMC-BH has a significant antitumor activity in GSCs, and that it induces GSC apoptosis by activating the JNK/ERK pathway.

## RESULTS

### DMC-BH inhibits proliferation and induces apoptosis in GSCs

**Figure 1:**
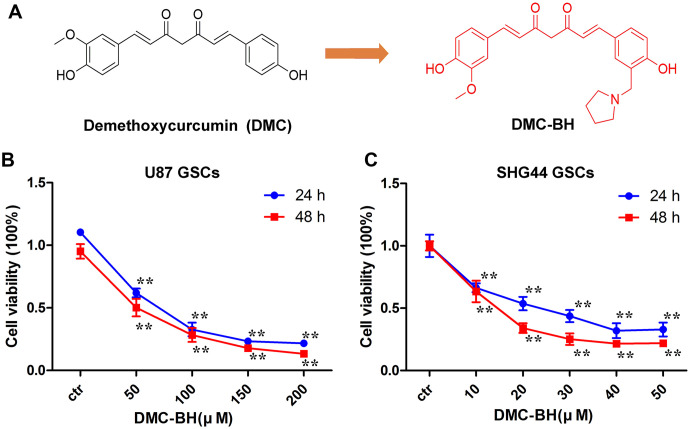
**DMC-BH inhibits proliferation of GSCs**. (**A**) Chemical structures of DMC-BH and DMC. (**B**, **C**) Effect of DMC-BH on proliferation of U87 and SHG44 cells in vitro; n =3.

To investigate whether DMC-BH (Figure 1A) inhibits proliferation of GSCs, we analyzed in vitro growth of U87 and SHG44 GSCs incubated with DMC-BH for 24 and 48 h, using MTT assay. As shown in Figure 1B, 1C, DMC-BH significantly inhibited GSCs cell proliferation in a dose- and time dependent manner.

To determine whether the DMC-BH-mediated inhibition of GSCs proliferation was associated with increase-edapoptosis, we analyzed apoptosis using TUNEL staining assay and Annexin V-FITC/PI double staining in U87 and SHG44 GSCs. As shown in Figure 2A, DMC-BH significantly increased the number of TUNEL positive cells. Further, using the Annexin assay, DMC-BH significantly increased the apoptotic rates in U87 (21.37% ±6.11) and SHG44 (27.10% ±4.00) GSCs (Figure 2B). Western blot results demonstrated that cleaved caspase-3, the crucial apoptosis executor, was increased after 24 h DMC-BH exposure (Figure 2C). Furthermore, cleaved PARP (poly (ADP-ribose) polymerase), the substrate of caspase-3, was significantly increased in GSCs after DMC-BH exposure. In addition, expression of the anti-apoptotic protein Bcl-2 decreased in GSCs after DMC-BH exposure. The increased levels of cleaved caspase-3 and PARP were associated with significantly increased activities of caspase-8, -3, and -9 after DMC-BH exposure in GSCs (P<0.05)(Figure 2D).

**Figure 2:**
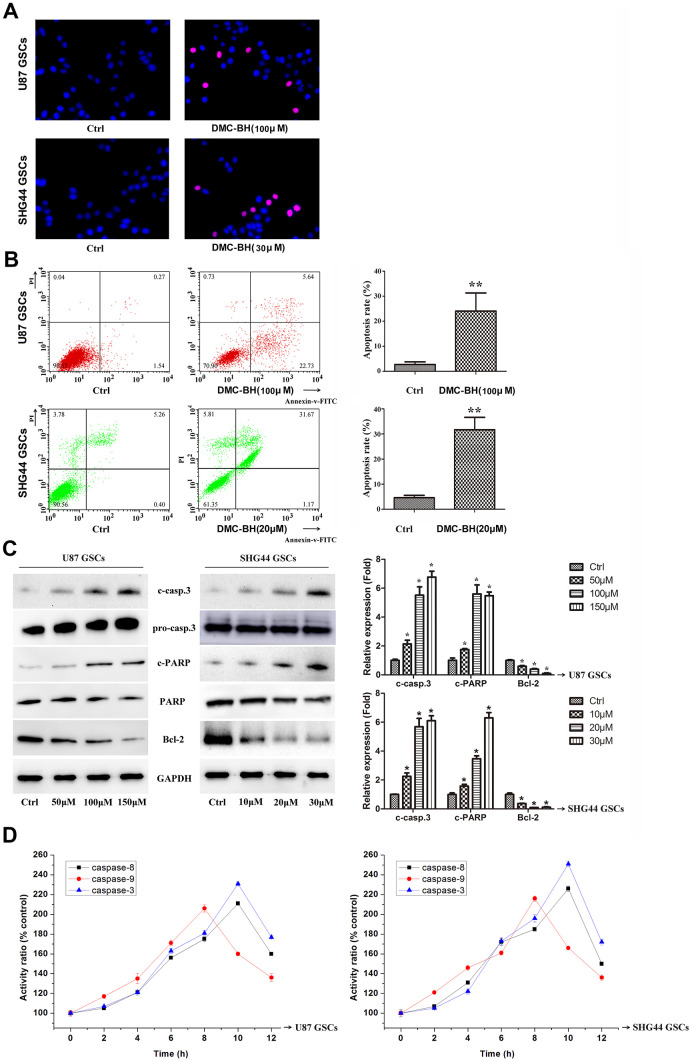
**DMC-BH induces apoptosis in GSCs.** (**A**) Morphological changes associated with apoptotic cell death analyzed by TUNEL staining. Red nuclear staining indicates apoptosis, while blue staining suggests normal nuclei. (**B**) Apoptosis analyzed using Annexin V-FITC/PI-staining flow cytometry. (**C**) Western analysis of apoptosis-related proteins caspase-3, PARP, and Bcl-2. (**D**) Caspase-3, -8 and -9 activity measured by ELISA.

**Figure 2:**
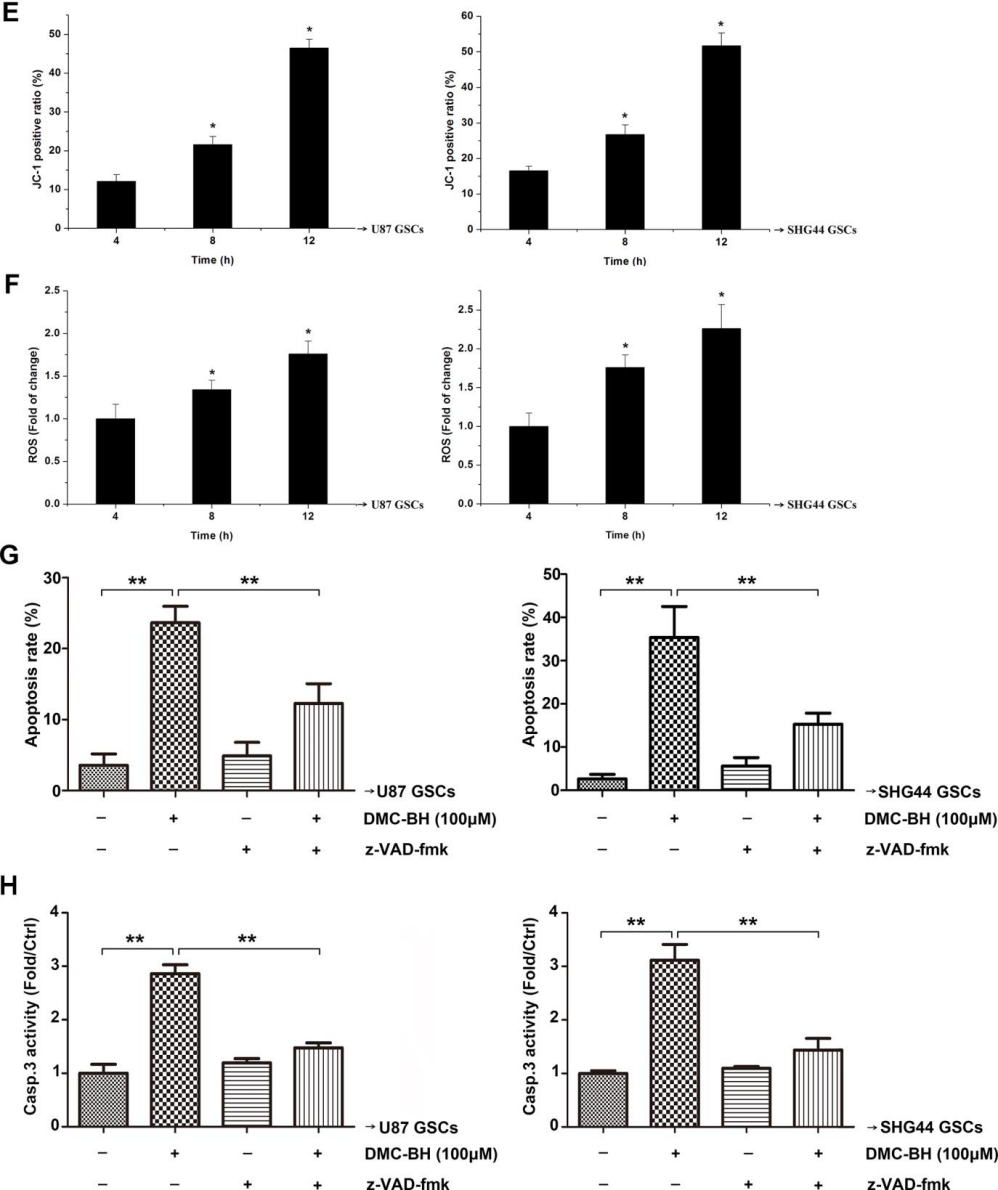
**DMC-BH induces apoptosis in GSCs.** (**E**) JC-1 positive cells analyzed by flow cytometry. (**F**) Intracellular ROS generation induced by 100 μM DMC-BH analyzed using DCFH-DA (10 μM) and flow cytometry. (**G**) Apoptosis of cells treated with DMC-BH and/or z-VAD-fmk for 24 h, analyzed by flow cytometry. (**H**) Caspase-3 activity measured using the substrate peptide Ac-DEVD-pNA; n =3.

Our results showed that 100 μM DMC-BH time-dependently decreased the mitochondrial transmembrane potential (MTP) in GSCs (Figure 2E). In addition, as shown in Figure 2F, ROS production increased after 100 μM DMC-BH treatment in U87 and SHG44 GSCs. To determine whether the DMC-BH-induced apoptosis was caspase-dependent, we used the pan-caspase inhibitor z-VAD-fmk. Our data showed that z-VAD-fmk could partially rescue the DMC-BH-induced apoptosis (Figure 2G), and reduce the DMC-BH-induced activation of caspase-3 in GSCs (Figure 2F). These results indicate that DMC-BH induces apoptosis in GSCs by the mitochondria dependent pathway.

### DMC-BH induces cell cycle arrest in GSCs

**Figure 3:**
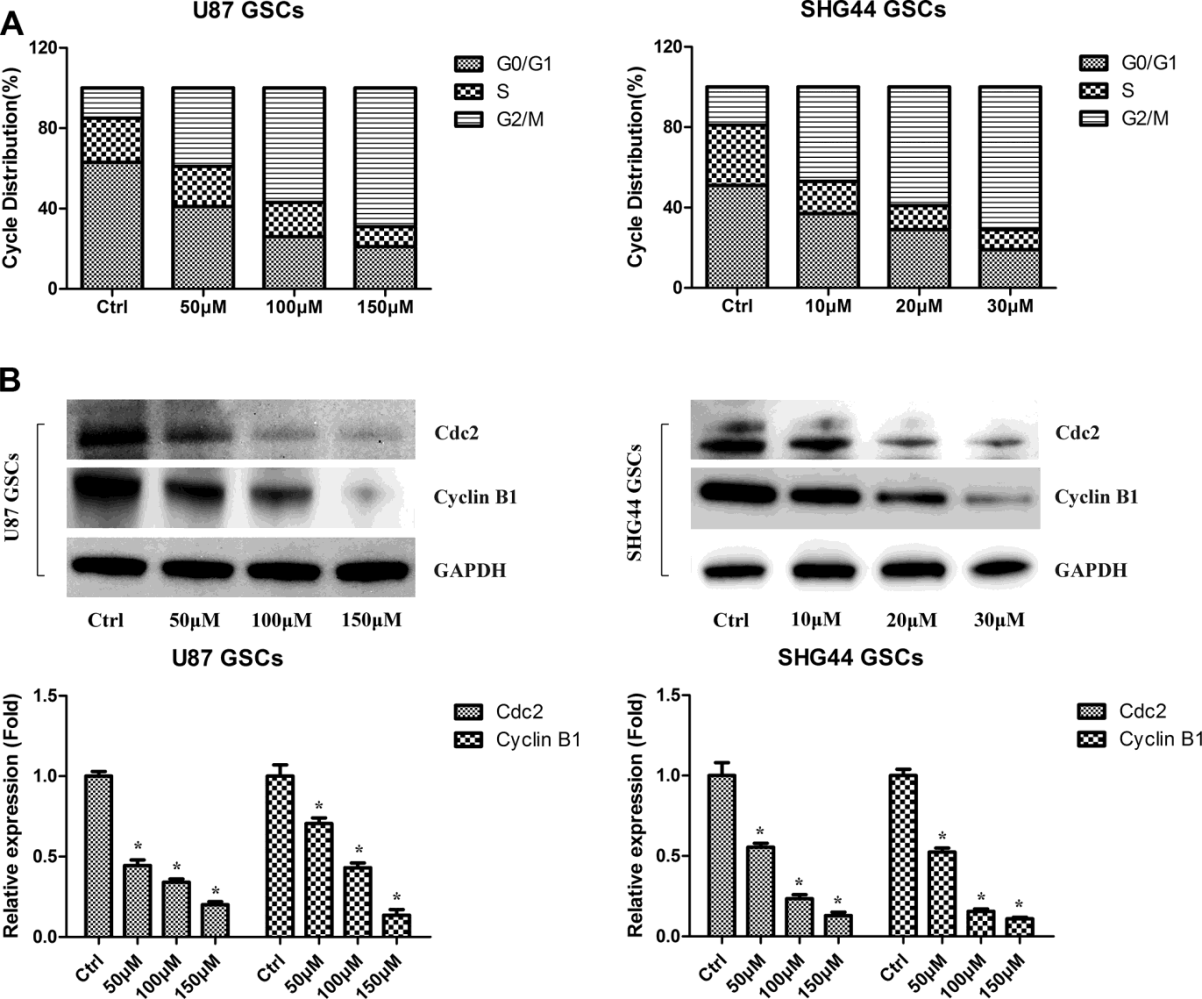
**DMC-BH induces cell cycle arrest in GSCs.** (**A**) PI flow cytometry analysis of DMC-BH-treated GSCs. (**B**) Western analysis of Cdc2 and cyclin B1 in DMC-BH-treated GSCs; n =3.

Next, we evaluated the effect of DMC-BH on cell cycle arrest of U87 and SHG44 GSCs. As shown in Figure 3A, increasing concentrations of DMC-BH dose-dependently increased the number of U87 and SHG44 cells in the G2/M phase. In addition, western blot analysis demonstrated that DMC-BH dose-dependently decreased the levels of cell cycle related proteins Cdc2 and Cyclin B1 in U87 and SHG44 cells (Figure 3B). Together, these results indicate that DMC-BH induces the G2/M cell cycle arrest in GSCs.

### DMC-BH-induced apoptosis in GSCs is mediated by JNK/ERK signaling

To examine which signaling pathway is activated in GSCs by DMC-BH, we used the Proteome Profiler Human Phospho-Kinase Array assay in U87 and SHG44 cells exposed to 20 and 100 μM DMC-BH for 6 h. As shown in Figure 4A, DMC-BH significantly increased phosphorylation levels of JNK, ERK and c-Jun in GSCs. The increased phosphorylation levels of p-Jun and p-ERK in DMC-BH-treated GSCs were also confirmed by western blotting (Figure 4B). In addition, DMC-BH increased the phosphorylation levels of JNK/ERK downstream proteins c-Jun and JunB in GSCs, indicating that DMC-BH activates the MAPK pathway in GSCs.

**Figure 4:**
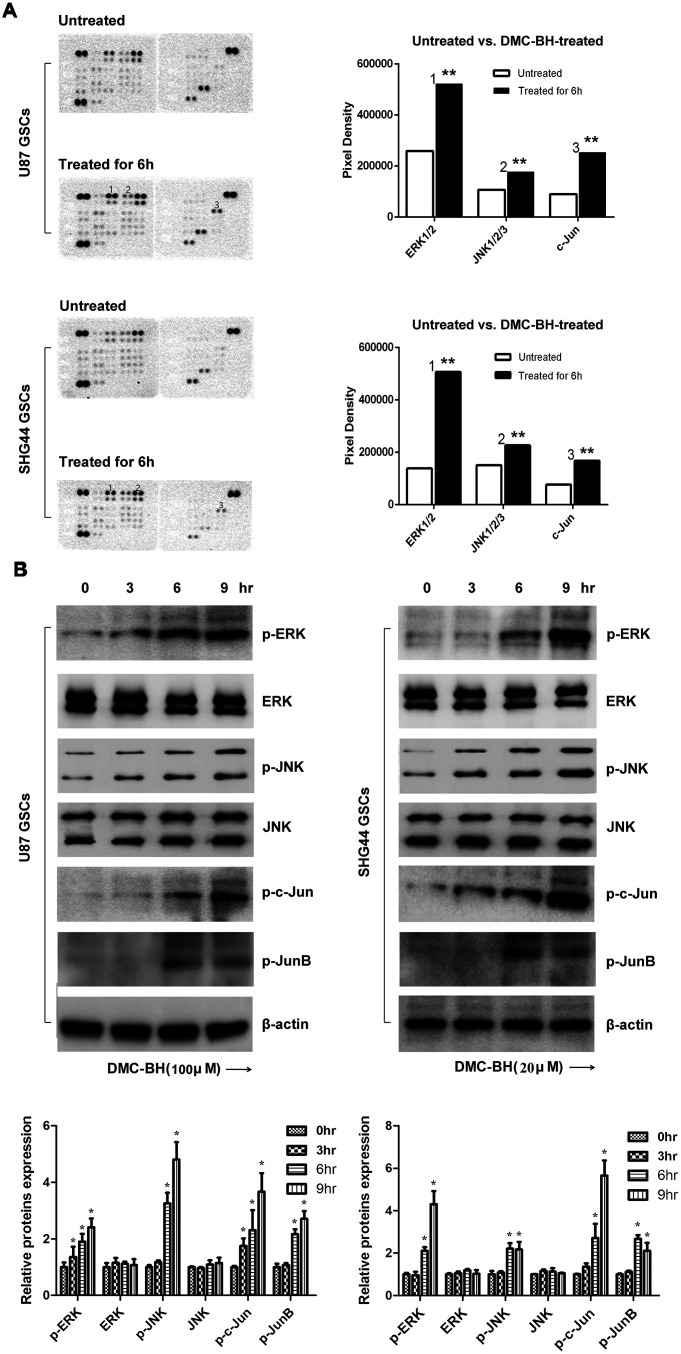
**DMC-BH-induced apoptosis in GSCs is mediated by JNK/ERK signaling.** (**A**) Phosphorylation protein levels in DMC-BH-treated GSCs analyzed by Proteome Profiler Human Phospho-Kinase Array. (**B**) Western analysis of phosphorylated levels of MAPK-related proteins p-ERK, p-JNK, p-c-Jun, and p-JunB in DMC-BH-treated GSCs.

**Figure 4:**
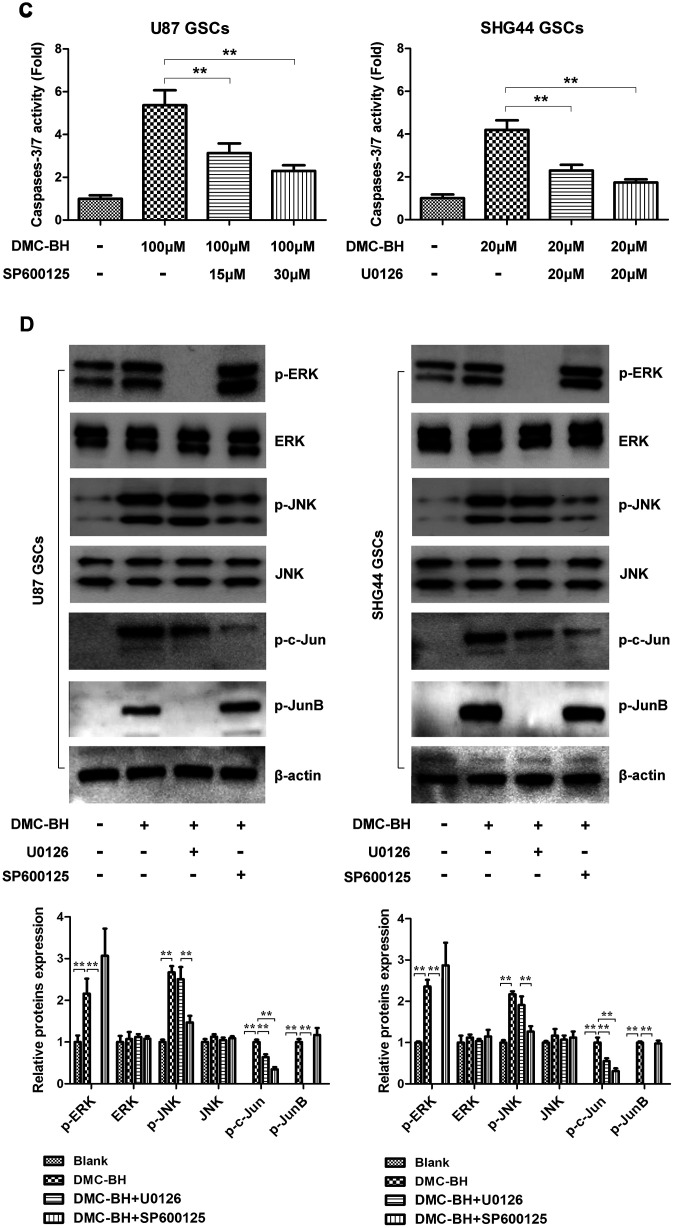
**DMC-BH-induced apoptosis in GSCs is mediated by JNK/ERK signaling.** (**C**) Caspase-3 activity measured after incubation with ERK inhibitor U0126 (20 μM) or JNK inhibitor SP600125 (30 μM). (**D**) Phosphorylated levels of MAPK-related proteins analyzed in DMC-BH-treated GSCs in the presence of U0126 (20 μM) or SP600125 (30 μM); n =3.

To determine whether activation of JNK and ERK regulates the DMC-BH-induced apoptosis in GSCs, we used the pharmacological inhibitors of JNK (SP600125) and ERK (U0126). As shown in Figure 4C, DMC-BH significantly increased the activities of caspases-3/7 in GSCs; however, their activation was sharply inhibited by SP600125 and U0126 treatment. Moreover, inhibition of JNK by SP600125 or ERK by U0126 resulted in reduced phosphorylation levels of p-JNK, p-ERK, p-c-Jun, and p-JunB (Figure 4D). Together, these results indicate that the DMC-BH-induced apoptosis in GSCs is mediated by the JNK/ERK signaling pathway.

### DMC-BH induces autophagy in GSCs

Autophagy, also known as type II programmed cell death, is a process by which cells degrade damaged organelles and macromolecules by lysosomes under the control of autophagy-related genes (Atg) [12]. Previous studies have shown that curcumin induces autophagy in GBM cells [13]. Considering that DMC-BH is a derivative of curcumin, we tested whether it induces autophagy in GSCs. As shown in Figure 5A, autophagosomes were clearly detectable by transmission electron microscopy in DMC-BH-exposed GSCs. Furthermore, DMC-BH increased the level of autophagy related protein LC3-II, but decreased the level of SQSTM1 in GSCs (Figure 5B). To confirm the effect of DMC-BH on autophagy in GSCs, GSCs viability was examined in the presence of autophagy inhibitor, 3-MA [14]. As shown in Figure 5C, 3-MA increased the levels of SQSTM1, but decreased the levels of LC3-II in DMC-BH-treated GSCs. As expected, inhibition of autophagy by 3-MA exacerbated the GSCs death (Figure 5D). In addition, the level of cleaved caspase-3 was increased in GSCs treated with DMC-BH and 3-MA compared to DMC-BH-treated GSCs (Figure 5E). Considering that autophagy is a double-edged sword in carcinogenesis, these data suggest that inhibition of autophagy drives DMC-BH-exposed GSCs toward apoptosis. Conversely, when apoptosis was blocked with the pan-caspase inhibitor z-VAD-fmk, autophagy was still observed under TEM, and U87 and SHG44 GSCs had increased LC3-II protein levels compared to GSCs treated with DMC-BH alone (P<0.05) (Figure 5F).

**Figure 5:**
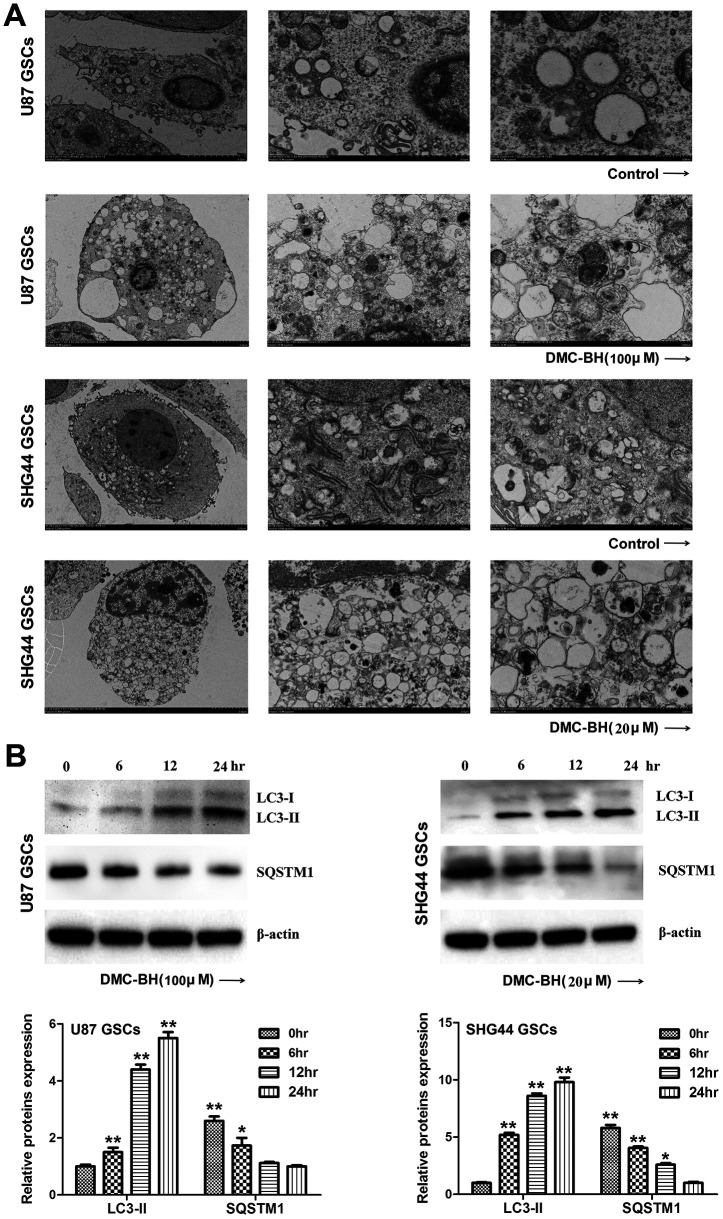
**DMC-BH induces autophagy in GSCs.** (**A**) Representative images of autophagosome formation in DMC-BH-treated U87 and SHG44 GSCs detected by TEM. (**B**) Levels of autophagy related proteins SQSTM1 and LC3 II measured in DMC-BH treated GSCs by western blotting.

**Figure 5:**
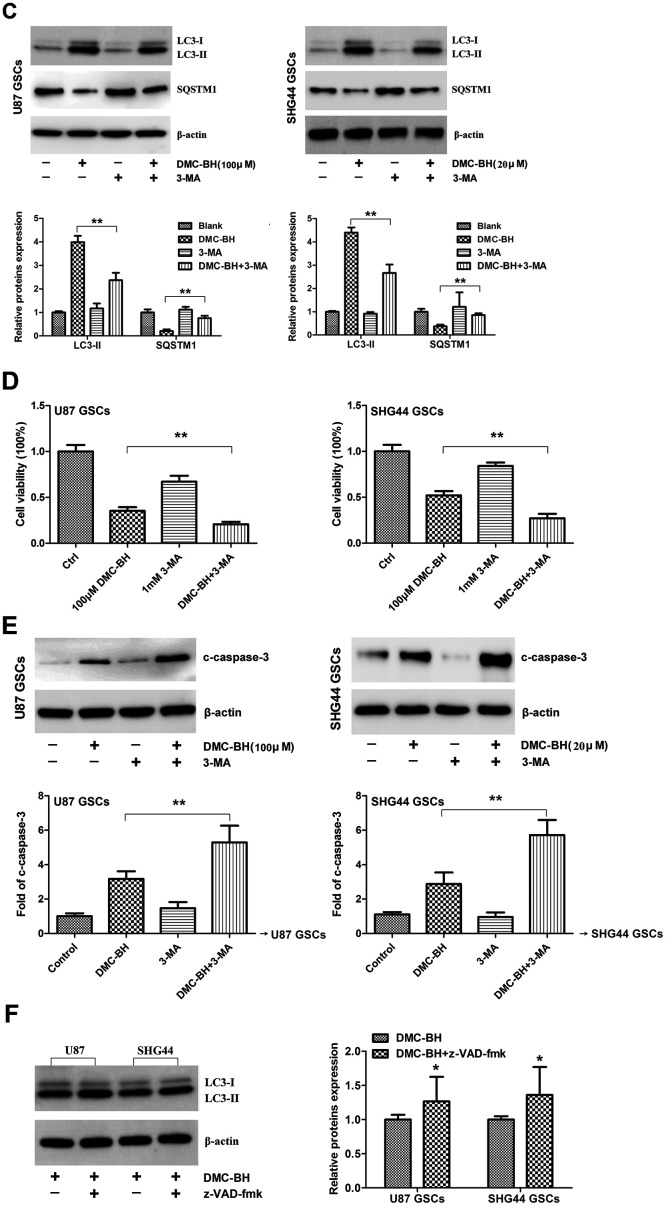
**DMC-BH induces autophagy in GSCs.** (**C**) SQSTM1 and LC3 II protein levels analyzed in DMC-BH treated GSCs in the presence of the autophagy inhibitor, 3-MA (1 mM; 3h) by western blotting. (**D**) Cell viability analyzed by MTT assay. (**E**) Western analysis of cleaved caspase-3 analyzed in GSCs incubated with DMC-BH +/- 3-MA by western blotting. (**F**) Western analysis of LC3 II protein levels in GSCs incubated with DMC-BH (100 μM and 20 μM) and/or z-VAD-fmk for 24 h; n =3.

### Acute toxicity of DMC-BH in mice

To evaluate the in vivo safety of DMC-BH, groups of ICR mice were injected intraperitoneally with a single dose of DMC-BH at 80, 64, 51.2, 40.96, and 32.77 mg/kg, or a vehicle control. As shown in Table 1, DMC-BH exhibited a somewhat increased acute toxicity in ICR mice; the calculated LD50 value was 48.0628 mg/kg. Thus, we selected a half dose of LD50 (20 mg/kg DMC-BH) for further experiments. No nude mice died under 20 mg/kg DMC-BH, indicating that 20 mg/kg DMC-BH was safe in nude mice.

**Table 1:** Acute toxicity of DMC-BH in mice.

**Group**	**Dose (mg/kg)**	**No. of mice**	**No. of dead mice**	**Mortality (%)**	**Dose logarithm**	**Probability unit (P + 5)**
1	80	10	9	90.0	1.903	6.280
2	64	10	7	70.0	1.806	5.520
3	51.2	10	5	50.0	1.709	5.000
4	40.96	10	4	40.0	1.612	4.750
5	32.77	10	2	20.0	1.515	4.160

LD50 =48.0628 mg/kg (43.6668~52.9014).

### DMC-BH inhibits GSCs intracranial orthotopic growth

In order to analyze the effectiveness of DMC-BH to inhibit GSCs intracranial growth, we performed intracranial orthotopic transplantation of tumor U87-luc and SHG44-luc GSCs xenografts. The mouse brain tumors were visualized by Bioluminescent Imaging (BLI) acquisition. As shown in Figure 6A, DMC-BH significantly inhibited the growth of GSCs in intracranial tumors compared with control PBS. BLI analysis of the intracranial tumors showed that the average tumor volume in DMC-BH-treated group was smaller than in PBS-treated group (Figure 6B). Further, immunohistochemical results demonstrated that DMC-BH treatment decreased expression of the glioma related proliferation marker Ki67 in GSCs xenografts (Figure 6C), and increased the number of TUNEL positive cells (Figure 6D). Western blot analysis showed that the levels of c-caspase-3, LC3-II, p62, p-ERK and p-JNK in DMC-BH-treated group markedly increased compared to the levels in PBS group (Figure 6E). No significant differences in body weight and mortality were observed between DMC-BH and PBS-treated groups (P>0.05) (Figure 6F). These results demonstrate that DMC-BH inhibits the in vivo growth of GSCs intracranial orthotopic tumor xenografts.

**Figure 6:**
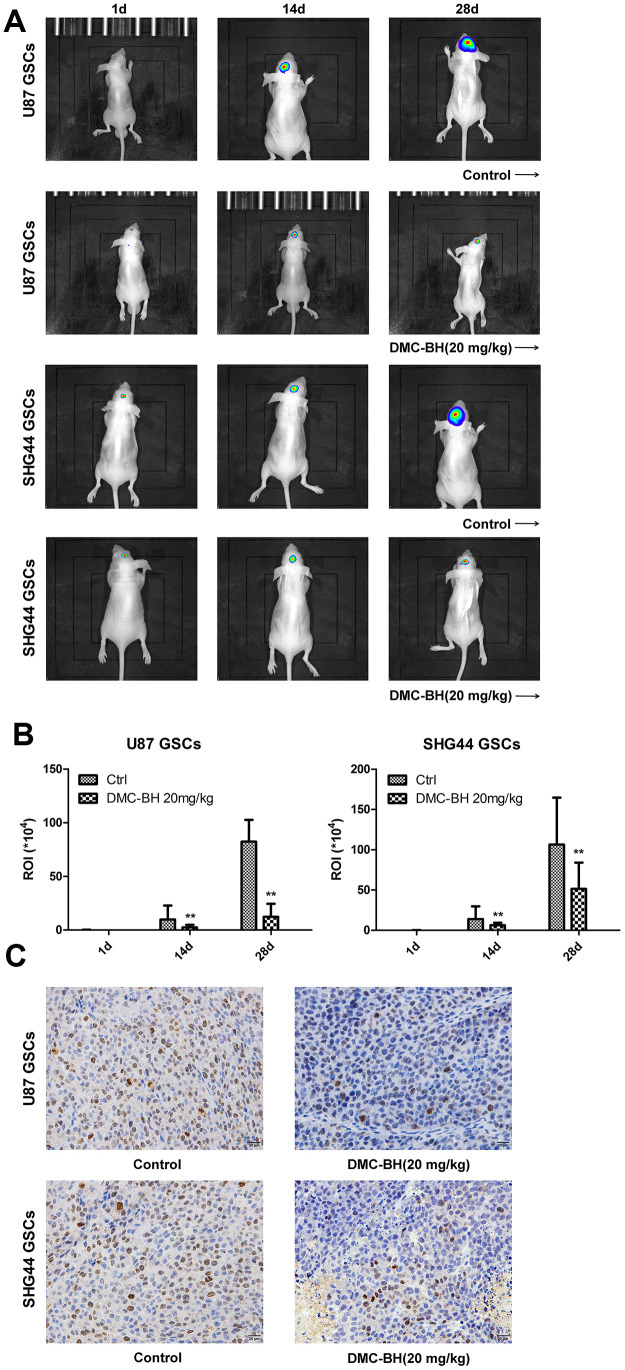
**DMC-BH reduces GSCs intracranial orthotopic growth.** (**A**) GSCs intracranial orthotopic growth after DMC-BH treatment measured by Bioluminescent Imaging (BLI). (**B**) The average tumor volume in intracranial analyzed by ROI recording. (**C**) Proliferation of intracranial tumor analyzed using the Ki67 biomarker.

**Figure 6:**
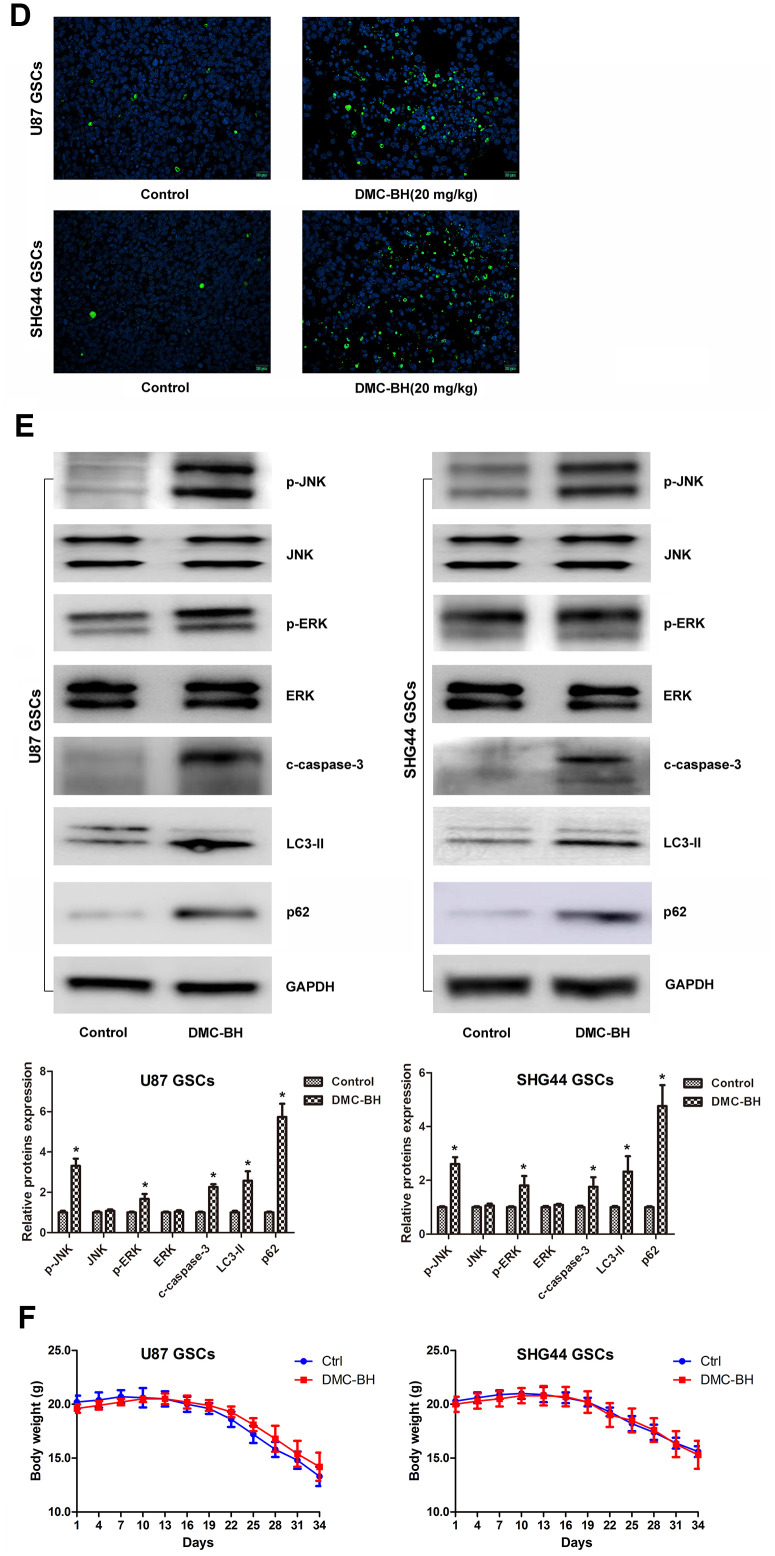
**DMC-BH reduces GSCs intracranial orthotopic growth.** (**D**) Apoptosis of intracranial tumor analyzed by TUNEL assay. (**E**) Western analysis of c-caspase-3, LC3-II, p-ERK and p-JNK levels in intracranial tumors. (**F**) Body weight loss in DMC-BH-treated and control mice; n =10.

## DISCUSSION

GBM is the most aggressive brain tumor characterized by fast progression and poor prognosis [16]. There are currently no effective treatments for GBM. Stem cells present in GBM are insensitive to radiotherapy and chemotherapy, and likely contribute to GBM treatment failure [17]. Although many new targets and novel drugs, such as anti-PD-1/PD-L1 therapy [18] and Bevacizumab [19] have emerged in recent years, the long-term effects for GBM patients are insignificant. Although previous studies have shown that demethoxycurcumin (DMC) can effectively inhibit glioma cell proliferation, it cannot effectively inhibit the growth of in situ intracranial tumors due to its inability to pass through the blood-brain barrier [6]. Temozolomide (TMZ), a first-line clinical drug for gliomas, can effectively cross the blood-brain barrier; however, it has a little inhibitory effect on glioma stem cells [20].

In this study, we have investigated the effect of DMC analogue, DMC-BH, on U87 and SHG44 GSCs in vitro, and on the growth of orthotopic xenograft tumors in vivo. Our results show that DMC-BH has potent anticancer effects against GSCs in vitro, and can effectively cross the blood-brain barrier to inhibit the growth of intracranial tumors in vivo. Our previous studies have shown that although DMC can inhibit subcutaneous tumors in nude mice, it has a little effect on intracranial orthotopic tumors, indicating that DMC cannot effectively pass through the blood-brain barrier [6]. Our current results demonstrate that DMC-BH is superior to DMC in glioma treatment, since it can cross the blood-brain barrier.

Our in vitro data showed that caspases were upregulated in DMC-BH-exposed GSCs, indicating that DMC-BH induces apoptosis of GSCs. This is supported by the previously described effect on upstream apoptotic proteins of Bcl-2 family and PARP [21]. Consistently, we observed the decreased Bcl-2 and increased cleaved PARP levels in U87 and SHG44 GSCs after DMC-BH treatment. The DMC-BH-induced apoptosis could partially be blocked by z-VAD-fmk, a caspase inhibitor. These data suggest that activation of caspase signaling is involved in DMC-BH-induced apoptosis of GSCs, although other apoptotic pathways may also be involved. Moreover, cell proliferation is often regulated through cell cycle [22]. Our results showed that DMC-BH induced G2/M phase cell cycle arrest in GSCs; this is consistent with a previous study demonstrating that DMC triggered G2/M cell cycle arrest in U87 MG glioma cells [23]. In addition, the DMC-BH induced cell cycle arrest was confirmed by the decreased expression of cell cycle related proteins Cdc2 and cyclin B1 in DMC-BH-exposed GSCs.

Autophagic cell death is another form of cell death, which is a programmed cell death that is different from apoptosis, and independent of caspases. Changes in autophagy activity have been associated with the development of several malignant tumors including gliomas [24]. Anti-apoptotic activity is an important cause of glioma resistance [25]. Studies have shown that autophagic cell death is an important step to overcome the apoptotic resistance [26]. Kanzawa et al. showed that TMZ induced autophagy and drug resistance in glioma cells; however, when combined with autophagy inhibitor bafiiomycin A1, the cells turned to apoptosis [27]. Yan et al. found that targeting autophagy sensitized glioma to TMZ treatment [28]. Both TMZ and curcumin inhibit glioblastoma growth; however, no synergy between curcumin and TMZ was found in inhibiting glioblastoma growth due to the induced autophagy [29], suggesting that autophagy blocked the apoptosis response. Electron microscopy is the only gold standard for detecting autophagy. Using transmission electron microscopy, our data demonstrated that DMC-BH induced autophagy in GSCs. LC3 protein, which is an important marker of autophagy, has two types, LC3-I and LC3-II; the LC3-I/II ratio reflects the autophagy activity of cells [30]. SQSTM1 (p62) is a ubiquitin binding protein involved in signal transduction, oxidative stress, and autophagy; it binds to autophage membrane protein LC3/Atg8 and transports protein polymers to the autophage [31]. Our western blot analysis demonstrated decreased SQSTM1 levels and increased LC3 II levels in DMC-BH-exposed GSCs. In addition, after blocking autophagy by 3-MA, DMC-BH-treated GSCs exhibited increased apoptosis and decreased viability, indicating that autophagy protects GSCs from DMC-BH-induced apoptosis.

Using Proteome Profiler Human Phospho-Kinase Array, our data showed abnormally elevated levels of p-JNK and p-ERK in GSCs after DMC-BH treatment, suggesting that DMC-BH might increase GSC apoptosis by activating ERK and JNK, which are involved in MAPKs signaling. MAPK is a family of important signal transducers; it consists of ERK, p38, JNK and ERK5 [32]. Our western analysis confirmed that DMC-BH increased the phosphorylation levels of ERK, JNK, c-Jun, and JunB in GSCs. JNK signal transduction pathway is an important branch of MAPK pathway, which induces apoptosis. Curcumin was shown to suppress retinoblastoma cell growth by regulating the JNK signaling [33]. In addition, increased activity of ERK1/2 has been associated with many human cancers, having both pro-apoptotic [34] and anti-apoptotic [35] functions. Our results showed that DMC-BH induced activation of ERK in GSCs, and that JNK and ERK inhibitors suppressed the DMC-BH-induced caspases-3/7 signaling, indicating that DMC-BH-induces apoptosis in GSCs by activating the ERK/JNK pathway.

In summary, our study shows that DMC-BH effectively inhibits proliferation of GSCs, and induces their apoptosis and autophagy. In addition, our data demonstrate that DMC-BH inhibits the growth of intracranial GSC tumors in vivo, indicating that it can cross the blood-brain barrier. The underlying mechanism of how DMC-BH inhibits proliferation and tumor growth of GSCs involves activation of the JNK/ERK pathway. These findings indicate that DMC-BH is superior to DMC in glioblastoma treatment.

## MATERIALS AND METHODS

### Reagents

DMC-BH was synthesized by China Pharmaceutical University. MTT cell proliferation kit (C0009) and Mitochondrial membrane potential assay kit with JC-1 (C2006) were purchased from Beyotime Biotechnology (Nanjing, China). TUNEL cell apoptosis in situ detection kit (KGA7062) was purchased from KeyGEN BioTECH (Nanjing, China). Annexin V-FITC/PI Apoptosis Detection Kit was obtained from Elabscience Biotechnology (Wuhan, China). All primary and secondary antibodies for western blotting were obtained from Abcam (Cambridge, UK). Caspase 3, 8, and 9 Activity Assay Kits were obtained from Beyotime Biotechnology (Nanjing, China). z-VAD-fmk, U0126 and 3-Methyladenine (3-MA) were obtained from Sigma-Aldrich (St. Louis, USA). PI and RNase solution were from BD Biosciences (San Jose, CA, USA). Proteome Profiler Human Phospho-Kinase Array (ARY003B) was obtained from R&D Systems (Minneapolis, Minnesota). SP600125 was obtained from AG Scientific, Inc. (San Diego, CA, USA).

### Cell culture

U87 and SHG44 cells were grown in Neural Stem Cell Basal Medium supplemented with B27, Penicillin-Streptomycin, Glutamine, Heparin, recombinant basic fibroblast growth factor, and recombinant epidermal growth factor (Cyagen, Santa Clara, CA 95050, USA), and were maintained in a humidified incubator containing 5% CO2 at 37 °C. Neural stem cell markers (nestin and CD133) were employed to identify GSCs.

### Cell proliferation assay

GSCs were plated at 5 x 103 cells per well in 96-well plates, and incubated with the indicated concentrations of DMC-BH for 72 h. Cell metabolic activity was analyzed by MTT Cell Proliferation and Cytotoxicity Assay Kit (Beyotime Biotechnology, Nanjing, China) according to manufacturer's instructions.

### Tunel staining apoptosis assay

GSCs were plated at 1×105 cells per well in 6-well plates, and treated with DMC-BH for 24- and 48-h. Cells were fixed with 4 % paraformaldehyde for 30 min, washed with PBS, and incubated in PBS containing 0.3% Triton X-100 for 5 minutes at room temperature. After that, GSCs were incubated with 50 μM TUNEL detection solution (Beyotime Biotechnology, Nanjing, China) and Hoechst 33,258 staining solution (Beyotime Biotechnology, Nanjing, China) for 10 min. The seal solution was quenched by fluorescence quenching and observed under fluorescence microscope. In this assay, the red nuclear staining indicates apoptosis, while the blue staining suggests normal nuclei.

### Annexin V/FITC apoptosis assay

2×105 cells per well were seeded in 6-well dishes, and incubated with DMC-BH for 24 and 48 hours. GSCs were stained with 5 μl Annexin V-FITC and 10 μl PI at room temperature for 10-20 minutes, centrifuged, and analyzed by flow cytometry.

### Mitochondrial transmembrane potential (MTP)

MTP was measured by flow cytometry; 5 μL of 10 μg/mL JC-1 solution was added into 500 μL of cell suspension, mixed, and incubated in dark for 20 min. Stained cells were analyzed by fluorescence activated cell sorting using the CXP analysis software (BD Biosciences, Franklin Lakes, NJ, USA).

### Western blot analysis

Whole cell extracts were prepared using RIPA lysis buffer (Beyotime Biotechnology, Nanjing, China). For tissue extracts, 0.1-0.2g of intracranial orthotopic tumor xenografts were lysed in a lysis buffer containing 20 mM Tris (pH 7.5), 150 mM NaCl, 1% Triton X-100, 2.5 mM sodium pyrophosphate, 1 mM EDTA, 1% Na3VO4, 0.5 μg/mL of leupeptin, and 1 mM PMSF, and homogenized with a motor homogenizer. Samples were centrifuged at 12,000 rpm for 15 min, and protein concentration was determined using a BCA protein Assay Kit (Beyotime Biotechnology, Nanjing, China). 50 μg protein/lane was resolved on a SurePAGE gel, and transferred to PVDF membrane. The membranes were incubated with primary antibodies against PARP-1 (Abcam, Shanghai, China), Bax (Abcam, Shanghai, China), Bcl-2 (Abcam, Shanghai, China), caspase-3 (Abcam, Shanghai, China), GAPDH ((Abcam, Shanghai, China)), JNK (Abcam, Shanghai, China), Phospho-JunB (Ser259) (Shanghai Abways Biotechnology Co.,Ltd., Shanghai, China), p-ERK (Shanghai Abways Biotechnology Co.,Ltd., Shanghai, China) and p-JNK (Abcam, Shanghai, China) at 4 °C overnight. After washing with TBST, the membranes were incubated with horseradish peroxidase (HRP)-conjugated secondary antibodies (Abcam, Shanghai, China) for 1 h, and the signal was detected with enhanced chemiluminescence (Amersham Life Science, Arlington Heights, IL).

### Caspase-3, 8 and 9 activity assays

Caspase activities were detected by using Caspase-3, 8 and 9 Activity Assay Kits (Beyotime Biotechnology, Nanjing, China) according to the manufacturer’s instructions. Briefly, cells were seeded in 96-well plates at a density of 5×104 cells/mL, and incubated with DMC-BH. Activity of caspase-3, 8, and 9 was measured in cell lysates using the Ac-DEVD-pNA substrate and a microplate reader (M200; TECAN, Japan) at 405 nm.

### Measurement of intracellular ROS production

The production of intracellular ROS was measured by using ROS Assay Kit (Beyotime Biotechnology, Nanjing, China). 2×105 cells per well were incubated in 6-well plates, and after incubation, the medium was aspirated, and intracellular ROS was detected using H2DCFDA. For flow cytometry, cells were obtained by centrifugation, washed with PBS, re-suspended in 500 μl of H2DCFDA (10 μM), and after 30 min incubation in the dark at 37°C, cells were immediately analyzed by flow cytometry.

### In vivo tumor model

80 male nude mice of 18-20g/6-8 weeks were randomly divided into a blank group (100 μl DMSO/PBS intraperitoneal (i.p.) injections) and DMC-BH group (20 mg/kg DMC-BH in 100 μl of DMSO/PBS intraperitoneal (i.p.) injection). Mice were placed in the laminar shelf for SPF, and the food, bedding, cage, and contact instruments were all used after high pressure disinfection. Amplified U87-luc or SHG44-luc GSCs were passaged in 6 cm culture dish in 5% CO2 incubator. 10% chloral hydrate (40 μL/10g) was used to anesthetize the mice. Mice were then fixed on stereotaxic apparatus under anesthesia, immobilized, and injected with U87-Luc cells; the concentration of cells was 1 × 108/mL. 5 × 105 U87-luc or SHG44-luc GSCs were then injected in the caudate nucleus of the nude mice through an infusion of 1 μL/min. The orthotopic glioblastoma growth was quantified by BLI using an IVIS SPECTRUM 200 system (Perkin Elmer). The drug administration started 10 days after intracranial implantation of U87-luc or SHG44-luc GSCs, and continued daily for 28 days. In vivo imaging confirmed that the tumors were formed; this was recorded as day 1. Control mice received an equivalent volume of physiologic solution each day. Animals were sacrificed by CO2 inhalation. The study was approved by the Institutional Animal Care and Use Committee of Affiliated Kunshan Hospital of Jiangsu University (Approval ID 2019-07-001). All operations were performed according to international guidelines concerning the care and treatment of experimental animals.

### Immunohistochemistry

Tissue sections were placed in xylene for 10 min and deparaffinized. After antigen thermal repair, endogenous peroxidase activity was inhibited, sections were blocked, washed, and incubated with a non-immune serum at room temperature for 10 min. Slides were then incubated with a primary antibody at 4 °C overnight, washed, and incubated with biotin-conjugated secondary antibody. Sections were counterstained with hematoxylin.

### Statistical analysis

All tests were performed using SPSS Graduate Pack 11.0 statistical software (SPSS, Chicago, IL). Descriptive statistics, including the mean and SE, and one-way ANOVA were used to determine significant differences. *, P < 0.05 and **, P <0.01 were considered statistically significant.
